# Herbal medicines for anorexia in lung cancer: A protocol for systematic review and meta-analysis

**DOI:** 10.1097/MD.0000000000023913

**Published:** 2020-12-24

**Authors:** Ju Ah Lee, Kyun Ha Kim, Geum Young Ko, Hwa-Seung Yoo, Jun-Yong Choi

**Affiliations:** a Hwapyeong Institute of Integrative Medicine; b National Clinical Research Center for Korean Medicine, Korean Medicine Hospital of Pusan National University; c East West Cancer Center, Seoul Korean Medicine Hospital of Daejeon University; d Department of Internal Medicine, Korean Medicine Hospital of Pusan National University; e School of Korean Medicine, Pusan National University, Republic of Korea.

**Keywords:** anorexia, herbal medicine, lung cancer, protocol, systematic review

## Abstract

**Introduction:**

Lung cancer is the leading cause of cancer-related death worldwide. Anorexia is the most common cause of malnutrition in lung cancer patients as well as an independent prognostic factor for cancer survival. This review will deal with the clinical evidence of herbal medicine use for reducing anorexia in lung cancer patients.

**Methods and analysis:**

Fourteen electronic databases will be searched from inception until October 2020. We will include randomized controlled trials (RCTs) assessing herbal medicines for anorexia in lung cancer patients. Interventions of any herbal medicines will be included. The methodological qualities of the included RCTs will be assessed via the Cochrane Collaboration tool for assessing the risk of bias. The Grading of Recommendations Assessment, Development, and Evaluation (GRADE) instrument will be used to evaluate the confidence in the cumulative evidence.

**Ethics and dissemination:**

This systematic literature review does not require an ethics review. This review will be published in a peer-reviewed journal and disseminated electronically and in print. The review will be updated to inform and guide healthcare practices.

**Registration number:**

reviewregistry1038.

## 1 Introduction

The leading cause of cancer-related death is lung cancer, and its prevalence is substantial.^[1]^ The World Health Organization stated that lung cancer's death rates would continue to rise, mainly due to increased global tobacco use.^[2,3]^ Malnutrition significantly impacts the prognosis of lung cancer patients and is prevalent in advanced lung cancer patients undergoing various anti-cancer therapy, such as cytotoxic chemotherapy and targeted therapies.^[4–6]^ The most common cause of malnutrition in lung cancer patients is anorexia, that is, the loss of appetite.^[7]^

There are many causes of anorexia in cancer, and the increase of proinflammatory cytokines and/or lactate are significant ones. These factors modulate the central nervous system's neurotransmitter cascades that can induce appetite loss in cancer patients.^[8]^

Anorexia alone is an independent prognostic factor for cancer survival.^[9]^ There are currently 2 drugs with clinical evidence of reducing anorexia in cancer patients: megestrol acetate (a progesterone derivative) and glucocorticoids.^[10–13]^ However, these drugs have significant adverse events. In patients receiving megestrol acetate, edema, thromboembolic phenomena, and deaths are frequently observed in patients.^[12]^ Patients on glucocorticoids have a higher risk for venous thromboembolism than those taking megestrol.^[14]^ Anorexia also impacts lung cancer patients’ daily quality of life (QoL) in their daily lives. Therefore, treatments for cancer-associated anorexia should be evaluated for impact on both QoL and survival.^[15]^

Cancer patients need safe and effective treatments for cancer-associated anorexia, such as herbal medicine.^[16]^ Traditional Asian medicine has treated the general loss of appetite for a long time. There are several clinical studies that compared traditional Asian medicine to megestrol acetate,^[17]^ and compared herbal medicine plus chemotherapy with chemotherapy alone,^[18]^ both of which showed promising results regarding anorexia in lung cancer patients.

This review will focus on anorexia in lung cancer patients from RCTs of herbal medicine for the evidence-based clinical practice for them.

## 2 Methods

### 2.1 Study registration

The proposal for this protocol complies with Preferred Reporting Items for Systematic Reviews and Meta-Analysis Protocols (PRISMA-P).^[19]^ With regard to the present systematic review, the protocol is registered in the ResearchRegistry with reviewregistry1038 being the unique identifying number. The present study adheres to the guidelines mentioned in the PRISMA statement concerning healthcare interventions’ meta-analyses.^[20]^

### 2.2 Ethical approval

Since this is not a clinical study, it does not require ethical approval.

### 2.3 Data sources

Fourteen databases would be searched from inception till the existing date: AMED, MEDLINE, Embase, the Cochrane Central Register of Controlled Trials (CENTRAL), as well as CINAHL. In addition, 6 Korea-based medical databases would also be searched. These include the Korean Traditional Knowledge Portal, OASIS, KoreaMed, the Korean Studies Information Service System, DBPIA, and the Korean Medical Database. Search will also be undertaken on 3 Chinese databases: The China Doctoral Dissertations and Masters’ Theses Full-text Database, CNKI (including the China Academic Journal, the China Proceedings of Conference Full-Text Database and the Century Journal Project), VIP, and Wanfang. Finally, a Japanese database would also be searched along with non-electronic searches of conference proceedings, 9 traditional Korean medicine journals, and our article files. The search strategy with respect to MEDLINE database is shown in Supplement 1. Similar search strategies would be adopted in other databases as well.

### 2.4 Types of studies

Quasi-RCTs and Prospective RCTs examining the efficiency of herbal medicine for anorexia among patients of lung cancer would find inclusion in the review. Treatments involving only herbal medicine and a combination of herbal medicine and another therapy will be taken into consideration. Both groups should have been equally administered any other treatment. Trials that entail a control intervention would also find inclusion. Typically, publication language would not be a restriction. Every study would be read in entirety. In case of disagreements about an article's inclusion, a discussion would be undertaken between all authors to resolve the issue. In the event such a resolution is not possible, the final decision will be made by an arbiter (JYC).

### 2.5 Types of participants

Lung cancer patients who are suffering from anorexia.

### 2.6 Types of interventions

Inclusion eligibility include interventions of any kinds of formulation (tablets, pills, capsules, extracts, powders, decoctions, injections, etc.) of herbal medicine. The present study would encompass studies using herbal medicines that have been prescribed by conventional East Asian medicine doctors. A review would be undertaken for the interventions’ compositions.

### 2.7 Data extraction

All articles’ hard copies would be derived and read in entirety. Two authors, namely, KHK and JAL would get the data extracted and examine the quality via an independent preformed data extraction form.

Moreover, all interventions involving acupuncture would be administered in compliance with the Standards for Reporting Interventions in Clinical Trials of Acupuncture (STRICTA).

### 2.8 Data collection and synthesis

#### 2.8.1 Outcome measures.

##### 2.8.1.1 Primary outcome

The primary outcome will be the improvement of anorexia.

##### 2.8.1.2 Secondary outcomes

Safety, the evaluation of which would be premised on adverse events and QoL changes, would be the secondary outcomes.

#### 2.8.2 Assessment of bias in the included studies

Cochrane Collaboration risk of bias assessment tool (version 5.1.0) would be used to evaluate the risk of bias by assessing the presence of allocation concealment, generation of random sequence, participants/personnel blinding, outcome assessment blinding, selective reporting, outcome data's comprehensiveness and other bias sources.^[21]^ These assessments’ evaluations would be undertaken by using “L”, “U”, and “H” indicating a low bias risk, an uncertain bias risk, and a high bias risk, respectively. A discussion involving all authors would be carried out to resolve disagreements, if any. When this resolution is not possible, the final decision will be made by an arbiter (JYC).

#### 2.8.3 Data synthesis

The 95% confidence interval (CI) and mean difference (MD) will gauge the impact of treatment in the form of continuous data. Forms of data would be converted into the MD. Both standard MD (SMD) and 95% CI would be utilized for outcome variables across various scales. With regard to dichotomous data, treatment effects would be presented as 95% CI and relative risk (RR). All statistical assessments would be carried out using the Review Manager (RevMan) version 5.4 for Windows (The Nordic Cochrane Center, Copenhagen, and the Cochrane Collaboration, 2012). In addition to contacting corresponding authors of studies in the case of missing information, we will also pool data across studies to conduct a meta-analysis using random/fixed effects models. We would utilize Cochrane Systematic Reviews’ GRADEpro software from for creating a summary of findings table.

#### 2.8.4 Unit of analysis issues

As far as crossover trials are concerned, we will use data from the initial treatment period. With regard to trials assessing more than one control group, primary analysis would entail the combination of data sourced from all control groups. The control groups’ subgroup analyses will be undertaken. Each patient would be counted on just one occasion during these analyses

#### 2.8.5 Addressing missing data

We will also perform intention-to-treat evaluations which encompass all randomized patients. With regard to patients with missing outcome data, we will carry out the last observation analysis (carried forward). In scenarios where there is availability of individual patient data, a review of the source or published trial reports would be undertaken to derive these data.

#### 2.8.6 Assessment of heterogeneity

Fixed or random effects models would be used to carry out the meta-analysis on the basis of our data analyses. With respect to the included studies’ heterogeneity, *I*^2^ and Chi-Squared tests will be utilized for evaluating along with *I*^2^ > 50%, which indicates a high level of heterogeneity. Upon observing heterogeneity, we will conduct subgroup analysis for exploring the possible reasons behind the same.^[21]^

#### 2.8.7 Assessment of reporting biases

We will generate funnel plots for detecting reporting biases during the availability of a minimum of 10 trials.^[22]^ Nevertheless, since funnel plot asymmetry cannot be deemed to draw an equivalence with publication bias, possible causes for asymmetry would be identified in the chosen studies. This includes true heterogeneity, small-study effects, and poor methodological quality.^[22,23]^

## 3 Discussion

Anorexia is an important symptom that can affect the QoL of patients with lung cancer. Although there has been a systematic review assessing the effect of Chinese herbal medicine on the QoL of patients with non-small cell lung cancer that showed improvement in QoL of such patients on administration of herbal medicine combined with chemotherapy, only overall QoL was evaluated and sub-components of the QoL (e.g., anorexia) could not be found.^[24]^

In summary, this review focused on the symptom of anorexia in patients of lung cancer patients included in RCTs conducted to evaluate evidence-based clinical practice for herbal medicine.

## Author contributions

JYC and JAL conceived the study, developed the study criteria, searched the literature, analyzed the data. JAL and KHK wrote the protocol. GYK and HSY conducted the preliminary search. JYC supervised the study protocol structure. All authors have read and approved the final manuscript.

**Conceptualization:** Ju Ah Lee KMD, Jun-Yong Choi.

**Investigation:** Geum Young Ko, Hwa-Seung Yoo.

**Methodology:** Kyun Ha Kim KMD, Hwa-Seung Yoo.

**Project administration:** Jun-Yong Choi.

**Writing – original draft:** Ju Ah Lee KMD, Kyun Ha Kim KMD.

## Supplementary Material

Supplemental Digital Content
