# Supplementary material for: Herbal medicines for anorexia in lung cancer: A protocol for systematic review and meta-analysis
Source: Medicine (Baltimore). 2020 Dec 24;99(52):e23913. doi: 10.1097/MD.0000000000023913 (PMC7769363; doi:10.1097/MD.0000000000023913)
Supplement: Supplemental Digital Content [file medi-99-e23913-s001.pdf]

Supplement 1. Search strategy for the MEDLINE database

#1 Search ("lung cancer cancer"[Mesh])

#2 Search ("lung carcinoma"[Mesh])

#3 Search (#1 OR #2)

#4 Search "Chinese medicine"[Mesh]

#5 Search "Kampo medicine"

#6 Search "Korean medicine"OR "Traditional Korean medicine"

#7 Search ((Plant Extracts"[tiab]) OR "Herbal Medicine"[tiab] or "herbal*"))
OR (("Plant Extracts"[Mesh]) OR "Herbal Medicine"[Mesh]

#8 #3 OR #4 OR #5 OR # 7

#9 #2 AND #8
